# Transmission of *Strongyloides stercoralis* Through Transplantation of Solid Organs — Pennsylvania, 2012

**Published:** 2013-04-12

**Authors:** Anjum Hasan, Marie Le, Jessica Pasko, Karen A. Ravin, Heather Clauss, Richard Hasz, Elizabeth A. Hunt, Elizabeth Bosserman, Isabel McAuliffe, Susan P. Montgomery, Matthew J. Kuehnert, Susan N. Hocevar, Francisca Abanyie

**Affiliations:** Geisinger Medical Center; Temple Univ Hospital; Gift of Life Donor Program; Pennsylvania Dept of Health; Div of Parasitic Diseases and Malaria, Center for Global Health; Div of Healthcare Quality Promotion, National Center for Emerging and Zoonotic Infectious Diseases; EIS Officer, CDC

*Strongyloides stercoralis* is an intestinal nematode endemic in the tropics and subtropics. Immunocompetent hosts typically are asymptomatic, despite chronic *Strongyloides* infection. In contrast, immunocompromised patients are at risk for hyperinfection syndrome and disseminated disease, with a fatality rate >50% (1–3). The infection source for immunocompromised patients, such as solid organ transplant recipients, is not always apparent and might result from reactivation of chronic infection after initiation of immunosuppressive therapy or transmission from the donor. In October 2012, the United Network for Organ Sharing (UNOS) notified CDC of a left kidney and pancreas recipient in Pennsylvania diagnosed with strongyloidiasis. This report summarizes the results of the investigation of the source of *Strongyloides* infection in three of four organ recipients. Testing of pretransplant donor and recipient sera confirmed that infection in the recipients was donor derived. This investigation underscores the importance of prompt communication between organ procurement organizations, transplant centers, and public health authorities to prevent adverse events in recipients when transmission is suspected. Additionally, it emphasizes the utility of stored pretransplant samples for investigation of suspected transplant-transmitted infections and the need to consider the risk for *Strongyloides* infection in organ donors.

## Case Investigation

On October 4, 2012, UNOS notified CDC of a left kidney and pancreas transplant recipient diagnosed with strongyloidiasis. UNOS also identified three additional organ recipients: the right kidney recipient, who received his transplant at the same institution as the index case; the liver recipient, who died within a few days after the transplantation; and the heart recipient, who was diagnosed with suspected reactivation of chronic strongyloidiasis 2 weeks earlier. CDC requested stored pretransplant serum from all organ recipients, along with stored donor serum for testing, to determine if infection with *Strongyloides* in the recipients was donor derived or reactivation of chronic infection. Evaluation of these specimens revealed that no recipient had detectable *Strongyloides* antibody before transplantation, but the donor had evidence of chronic infection based on positive serologic results.

### Organ donor

In July 2012, a Puerto Rico-born Hispanic man, aged 24 years, was admitted to a local emergency department with multiple gunshot wounds. After a 9-day hospitalization, he died, and his heart, kidneys, pancreas, and liver were transplanted into four recipients the next day. History obtained from his mother indicated that the donor was a healthy young male who often visited Puerto Rico. *Strongyloides* infection risk was not considered; therefore, testing was not performed before organ recovery.

### Kidney and pancreas recipient

This recipient is a U.S.-born white man, aged 64 years, with end-stage renal disease secondary to long-standing diabetes mellitus who had never traveled outside the United States. Nine weeks posttransplant, he developed severe nausea, anorexia, and abdominal distention and was admitted to the hospital. Stool studies and biopsies performed during an esophagogastroduodenoscopy revealed *S. stercoralis* adult worms; larvae were found in urine studies. The patient was treated with ivermectin and albendazole, and after a hospitalization complicated by *Enterobacter cloacae* bacteremia, periduodenal abscess, and loss of pancreatic transplant function, he was discharged in stable condition on ivermectin. Repeat stool analyses were negative 3 days after starting therapy.

### Kidney recipient

This recipient is a U.S.-born adolescent, aged 14 years, with end-stage renal disease as a result of a single dysplastic kidney; he had never traveled outside the United States. He was contacted for evaluation 10 weeks posttransplant, after the left kidney and pancreas recipient received a diagnosis of strongyloidiasis. He was discovered to be ill with fever, rash, malaise, anorexia, nausea, vomiting, and diarrhea. He was diagnosed with strongyloidiasis via esophagogastroduodenoscopy-obtained biopsy and stool testing. He was treated with ivermectin for 4 weeks and albendazole for 2 weeks. Repeat stool specimens were negative 3 days after starting therapy and remained negative as of November 2012.

### Liver recipient

This recipient was a Hispanic man, aged 66 years, with a history of hepatic failure secondary to chronic hepatitis C infection. He tolerated surgery and was clinically stable until postoperative day 4, when his heart stopped and he was unresponsive to attempts at resuscitation. At autopsy, no evidence of *Strongyloides* infection was found; cause of death was undetermined.

### Heart recipient

This recipient was a U.S.-born Hispanic man, aged 59 years, with ischemic cardiomyopathy; he lived in Puerto Rico for 6 months as a teenager. He remained clinically stable posttransplant and was discharged 11 days after surgery. He experienced multiple episodes of organ rejection and was treated with high doses of steroids. Seven weeks posttransplant, he was readmitted to the hospital with fever and a respiratory illness and required intubation in response to rapid decompensation. He was diagnosed with a viral respiratory illness and given oseltamivir and antibiotic and antifungal medications. A bronchoscopy performed on hospital day 3 showed *S. stercoralis* larvae. He was started on ivermectin and albendazole for treatment of suspected reactivated chronic strongyloidiasis. He developed gram-negative and enterococcal bacteremia and vancomycin-resistant enterococcal meningitis and became neurologically compromised. Life support was withdrawn, and he died 11 weeks posttransplant.

### Editorial Note

Most *Strongyloides* infections in organ transplant recipients are thought to be caused by reactivation of chronic infection after initiation of immunosuppressive therapy. Donor-derived infection has been reported, but the incidence of transmission is unknown (4,5). During 2009–2012, CDC assisted in seven investigations of organ donors and associated recipients with strongyloidiasis determined to be donor derived. Donor-derived infection is difficult to prove, especially if the infected recipient is from a region in which *Strongyloides* is endemic. Archived pretransplant serum samples were available for recipient testing in this investigation. Results of that testing contributed to the determination that infection was donor derived and not reactivated chronic infection in the recipients.

This investigation revealed several gaps in current understanding and assessment of the risk for transplant-transmitted strongyloidiasis. Specific recommendations are lacking for *Strongyloides* testing of organ donors from areas in which it is endemic. The parasitic infections sections of the American Society for Transplantation’s guidelines for screening prior to solid organ transplantation recommend testing donors and recipients for *Toxoplasma* and *Trypanosoma cruzi* (the cause of Chagas disease), but only recommend screening for *Strongyloides* in recipients from areas in which the nematodes are endemic, with no mention of donor screening (6,7). These guidelines are not policy, thus screening of donors and recipients for parasitic infections is voluntary, resulting in varied practices among organ procurement organizations and transplant centers based on the perceived risk in their respective patient populations. The growing evidence of transplant transmission of *Strongyloides*, reported here and in the recent literature, might support development of recommendations for specific testing of donors and recipients from endemic regions to prevent severe strongyloidiasis in recipients (1,4,5). A minimum of three serial stool examinations for larvae, using specialized concentration techniques, is the gold standard for diagnosis of *Strongyloides* infection, but this might not be feasible in patients who have poor gastrointestinal function or are brain dead. Tests to detect parasite-specific antibody, such as an enzyme-linked immunoassay, also are available and are valuable in identifying *Strongyloides* infection (8). If infection is confirmed in the donor, prophylaxis could be given to recipients to avert adverse outcomes.

What is already known on this topic?

*Strongyloides* infections in organ transplant recipients are thought to be caused mainly by reactivation of chronic infection after initiation of immunosuppressive therapy, which can lead to hyperinfection or disseminated disease. The American Society for Transplantation’s guidelines are in place to screen solid organ transplant recipients, but not donors, to assess the risk for reactivation of chronic infection in those from areas in which *Strongyloides* is endemic.

What is added by this report?

Donor-derived *Strongyloides* infection might be more common than previously believed. In these investigations, a single donor was the source of infection for three of four organ recipients. Testing of pretransplant serum contributed to the determination that infection was donor derived.

What are the implications for public health practice?

Screening of donors from *Strongyloides*-endemic areas might help to protect organ recipients. Rapid communication among transplant centers and organ procurement organizations is vital to protect the health of organ recipients when potential transmission of disease or medical conditions from the donor is a concern.

Rapid communication among transplant centers with patients who received organs from a single donor also is essential. The Organ Procurement and Transplant Network encourages organ procurement organizations and transplant programs to communicate promptly through its Patient Safety System, especially when there is concern for potential transmission of disease or medical conditions to the organ recipient from the donor. Such communication ideally should occur within 24 hours after knowledge of or concern for transmission, because multiple recipients might be adversely affected (9).

This investigation illuminates two gaps that need to be filled to improve transplant safety in solid organ recipients at risk for *Strongyloides* infection: 1) developing recommendations for screening of donors from *Strongyloides*-endemic areas, and 2) improving communication among transplant centers and organ procurement organizations. Advances in these areas might be life-saving for immunocompromised hosts.
